# Change in adaptive and maladaptive exercise and objective physical activity throughout CBT for individuals with eating disorders

**DOI:** 10.1007/s40519-023-01566-z

**Published:** 2023-04-20

**Authors:** Olivia Wons, Elizabeth Lampe, Anna Gabrielle Patarinski, Katherine Schaumberg, Adrienne Juarascio

**Affiliations:** 1 Department of Psychological and Brain Sciences, Drexel University, Stratton Hall, 3141 Chestnut Street, Philadelphia, PA 19104 USA; 2 Center for Weight, Eating, and Lifestyle Science, Drexel University, Stratton Hall, 3141 Chestnut Street, Philadelphia, PA 19104 USA; 3 Department of Psychiatry, University of Wisconsin-Madison, 6001 Research Park Blvd, Madison, WI 53719 USA

**Keywords:** Maladaptive exercise, Adaptive exercise, Eating disorders, Objectively measured physical activity, Step count, Minutes of moderate-to-vigorous physical activity, Cognitive behavioral therapy

## Abstract

Maladaptive exercise (i.e., exercise that compensates for binge eating or is used to avoid negative consequences of not exercising-like weight gain) is a common eating disorder (ED) behavior, yet, some individuals with EDs only engage in adaptive exercise. CBT for EDs targets reducing maladaptive exercise but does not address adaptive exercise. Thus, research is limited on how adaptive and maladaptive exercise are impacted during CBT for EDs. The current study examined how assessor-rated adaptive and maladaptive exercise and objectively measured physical activity changed over a 12-week CBT treatment among adults with transdiagnostic binge eating and restrictive eating that did and did not engage in maladaptive exercise at the start of treatment (*n* = 13 non-maladaptive exercise group, *n* = 17 maladaptive exercise group). The overall amount of adaptive and maladaptive exercise was measured via the Eating Disorder Examination Interview and objectively measured physical activity (e.g., step count, minutes of moderate-to-vigorous physical activity [MVPA]) was measured via a wrist-worn fitness tracker. Throughout treatment, the overall amount of adaptive exercise did not significantly change for both groups, but the overall amount of maladaptive exercise significantly decreased in the maladaptive exercise group. Step count did not significantly change for both groups, but minutes of MVPA significantly increased over treatment for the non-maladaptive exercise group. Increases in step count and minutes of MVPA were not associated with ED symptom changes in either group. These results enhance an understanding of exercise changes during transdiagnostic CBT-based ED treatment for individuals with varying baseline exercise profiles.

*Level of evidence:* Level 1, randomized controlled trial

## Introduction

Maladaptive exercise is a prominent factor in the etiology, development, and maintenance of symptoms across binge-spectrum eating disorder (ED) diagnoses [1], with prevalence rates of nearly 55% among individuals with bulimia nervosa (BN; [2] and 21% binge eating disorder [3]. Current theoretical conceptualizations define Maladaptive exercise as either: (1) driven (e.g., exercise that is intense and has a compulsive quality)[1, 4], and/or (2) compensatory (e.g., exercise that is intended to compensate for the effects that food consumption has on shape and weight) [2, 5],E. C. [6]. Driven exercise is considered a compensatory behavior within diagnostic criteria for many EDs including BN [7]. While frequent compensatory behaviors preclude a diagnosis of BED, individuals can still meet diagnostic criteria for BED and engage in relatively small amounts of maladaptive exercise [3]. For example, an individual with BED may endorse engaging in small amounts of exercise instead of spending time with friends to avoid gaining weight (i.e., feeling driven/compelled to exercise). Alternatively, an individual with BED may endorse walking around the block after they experience a binge episode with the hopes of “burning off” some of the calories they consumed (i.e., exercising to compensate for binge eating).

Despite the documented negative effects of maladaptive exercise engagement among individuals with BN [8, 9],C. [10], a growing body of literature suggests even individuals with EDs can benefit from both overall, general physical activity (i.e., any movement of the body that uses energy) and structured exercise (i.e., structured physical activity undertaken for the primary purpose of getting exercise; [11–14]). For example, structured adaptive exercise (i.e., non-compensatory exercise, not engaged in with the sole intention to control shape or weight, and non-driven, beneficial associations including stress reduction and socialization) can reduce driven exercise behaviors, drive for thinness, binge eating frequency, compensatory behaviors, and body dissatisfaction [14–17]. Lack of clarity around the role of structured exercise in symptom presentation for individuals with EDs has led to uncertainty about how to address exercise during treatment for individuals with EDs, including individuals with transdiagnostic binge eating and restrictive eating for whom exercise may serve either a compensatory and/or compulsive function.

Cognitive Behavioral Therapy (CBT), the most widely researched treatment for EDs [18, 19], views maladaptive exercise as a behavior that needs to be reduced. The most widely used CBT manual [20] suggests encouraging adaptive exercise but provides limited and vague guidance for clinicians on how to do this. Specifically, Fairburn’s CBT manual states, “It is important to note that CBT-E does not discourage exercising, even in underweight patients. Getting in “good shape” psychologically and physically is integral to CBT-E, but patients may need help in learning how to exercise in a different and less extreme way; for example, by taking up a team sport or by exercising with friends. Social exercising is of general benefit too as it may enhance patients’ socializing, something that can be a problem, while also providing an opportunity for body exposure.” Due to CBT’s emphasis on reducing maladaptive forms of exercise, the majority of research to date on the impact of CBT on exercise behaviors has focused on changes in maladaptive exercise [21–23], with results broadly demonstrating that CBT, in an outpatient setting, is effective at reducing maladaptive exercise in individuals with BED [16, 17, 22–24], despite only vague instructions to target the symptom. No study to date has examined changes in both adaptive and maladaptive exercise behaviors and overall physical activity over the course of CBT.

To date, there remain three unanswered questions about how both overall physical activity and overall adaptive and maladaptive structured exercise separately change during CBT for transdiagnostic binge eating and restrictive eating, suggesting additional research is needed in this area. First, no study to date has used a multimodal assessment approach to separately assess the amount of overall objectively measured physical activity (i.e., step count and minutes of moderate-to-vigorous physical activity (MVPA)) and the amount of maladaptive and adaptive exercise (i.e., duration and frequency of exercise episodes) throughout a CBT treatment for individuals with transdiagnostic binge eating and restrictive eating. A multimodal assessment of exercise shifts would inform understanding of whether objective physical activity changes. For example, perhaps pre-treatment bouts of maladaptive exercise are typically long (e.g., 60–90 min) and over the course of treatment become replaced with shorter, more frequent bouts of adaptive exercise. Alternatively, perhaps duration and frequency of adaptive and maladaptive exercise remains stable with only cognitions changing (e.g., the same duration of structured exercise is now engaged in for adaptive rather than maladaptive reasons). Second, no studies to date have examined differences in adaptive versus maladaptive exercise changes overall amount of physical activity among those with EDs who do and do not present treatment engaging in maladaptive exercise. It is possible that patterns of change in overall physical activity and adaptive exercise during CBT may differ for individuals who do and do not engage in maladaptive exercise at pre-treatment. Third, research to date [22, 23] has not tested whether changes in the amount of overall physical activity are associated with change in ED symptoms, limiting our ability to understand differential associations with treatment outcomes between overall physical activity, structured adaptive exercise, and structured maladaptive exercise and whether these associations differ based on whether participants are engaging in maladaptive exercise at pre-treatment.

Given extant literature reviewed above, the current study had four aims. First, to test the hypothesis that the amount of driven exercise, compensatory exercise, driven and compensatory exercise, and overall maladaptive exercise will significantly decrease from pre- to post-treatment among those who are engaging in maladaptive exercise at pre-treatment. It is hypothesized that the amount of driven exercise, compensatory exercise, driven and compensatory exercise, and overall maladaptive exercise will significantly decrease from pre- to post-treatment as extant research has largely demonstrated that CBT, in an outpatient setting, is effective at reducing maladaptive exercise in individuals with BED [16, 17, 22–24]. Second, to test whether there is an interaction between change in the amount of adaptive exercise among those who are and are not engaging in maladaptive exercise at pre-treatment. We hypothesized that there would be no significant change in the amount of adaptive exercise over the course of treatment between those that did and did not enter treatment engaging in maladaptive exercise. This hypothesis is driven by the notion that CBT treatment for eating disorders does not aim to increase adaptive exercise, and the CBT manual [20] provides only vague guidance for clinicians to promote adaptive exercise engagement in individuals with EDs. Third, to test whether there is an interaction between change in total objectively measured physical activity over the course of CBT between those who are and are not engaging in maladaptive exercise at pre-treatment. We hypothesized that step count and minutes of MVPA would significantly decrease for individuals who engaged in maladaptive exercise at the start of treatment compared to those that did not engage in maladaptive exercise at the start of treatment. This hypothesis is proposed on the basis that maladaptive exercise tends to reduce over the course of an outpatient CBT treatment, and thus, reduces an individual’s total amounts of physical activity [16, 17, 22–24]. Furthermore, there is no evidence to suggest the current CBT treatment supports individuals in maintaining their total amount of physical activity while also reducing maladaptive exercise. Finally, the current study aimed to test whether change in total objectively measured physical activity from pre-treatment to post-treatment was associated with change in ED symptoms over the course of CBT between those who did and did not engage in maladaptive exercise at the start of treatment. We hypothesized that greater improvements in ED symptoms would be associated with increased or stable step counts and minutes of MVPA among individuals who did not engage in maladaptive exercise at the beginning of treatment, and we had no specific directional hypothesis for individuals who engaged in maladaptive exercise at the beginning of treatment. It is hypothesized that greater improvements in ED symptoms would be associated with increased or stable step counts and minutes of MVPA among individuals who did not engage in maladaptive exercise at the beginning of treatment, because, broadly, non-maladaptive physical activity is associated with less ED symptoms [13].

## Methods

### Participants and recruitment

Participants (*n* = 30) were recruited via healthcare referrals, radio, newspaper, and online advertisements including social media. As part of a larger parent study, participants were recruited to participate in a study (ClinicalTrials.gov Identifier: NCT04126694; R43 MH121205**)** designed to test the feasibility and acceptability of a new sensor-based smartphone application when used as an adjunct to CBT-E focused version for adult patients with transdiagnostic binge eating and restrictive eating pathology. Participants were excluded if they were actively participating in a structured behavioral weight loss treatment. Inclusion criterion were that participants: engaged in 12 or more objectively large binge eating episodes within a 3-month period, engaged in three or more episodes of fasting for five or more waking hours per week in the last 4 weeks (dietary restriction), agreed to wear a fitness tracker every day for the duration of the study (12 weeks), were between the ages of 18–65, and had a BMI of 18.5–35.

### CBT treatment

All participants completed a 12-week CBT-E treatment. The CBT-E treatment instructed clinicians to address engagement in maladaptive exercise through psychoeducation and behavioral experiments. Based on the CBT-E treatment model, compensatory exercise was not explicitly targeted in treatment as reductions in binge episodes would reduce the need for engagement in compensatory behaviors broadly. Clinicians were instructed to address driven exercise similar to other compulsive behaviors, such as self-weighing and shape checking through behavioral experiments.

### Assessment procedures

The current study received Institutional Review Board approval. Data for the current study were collected at each assessment point and during weekly therapy sessions using a pre-session questionnaire. An initial phone screening was completed and then informed consent was collected at the pre-treatment assessment. All participants were provided with a wearable fitness tracker (the MiFit Smart Band) at the pre-treatment assessment and were instructed on how to use it. Participants were asked to wear the tracker throughout the entirety of the 12-week treatment and to charge it as necessary (during showers or a time when they were not exercising or sleeping). The two mid-treatment assessments occurred after session four and session eight and the post-treatment assessment occurred after session 12. All assessments were completed remotely due to the COVID-19 pandemic. Participants were sorted into two groups based how they responsed to questions about maladaptive exercise at the baseline assessment: 1) the non-maladaptive exercise group (*n* = 13) and 2) the maladaptive exercise group (*n* = 17). A participant was sorted into the maladaptive exercise group if they reported engaging in at least one maladaptive exercise episode throughout the 12 weeks prior to the baseline assessment.

### Measures

#### Modified eating disorder examination (EDE)

The current study used a modified version of the EDE to determine diagnosis and to assess whether participants were engaging in adaptive or maladaptive exercise at pre-treatment and post-treatment. The EDE is a semi-structured clinical interview assessing ED psychopathology [25]. The interview was modified by the researchers to separately assess adaptive and maladaptive exercise. In the modified EDE, assessors asked questions generated by our research team that separately assessed the duration and frequency of solely adaptive exercise episodes and the duration and frequency of all maladaptive exercise episodes, which included driven exercise only episodes, compensatory exercise only episodes, and combined driven and compensatory exercise episodes. Driven exercise was defined as any exercise episode that had a compulsive quality to it and was accompanied with a subjective sense of being out of control, compelled, rigid, or obligatory and resulted in functional impairment. Compensatory exercise was defined as an exercise episode that was intended to compensate for an objective or subjective binge eating episode. Exercise that was both driven and compensatory included maladaptive exercise episodes that had both a compulsive quality and were also intended to compensate for an objective or subjective binge episode. Because any individual maladaptive exercise episode could be either driven, compensatory, or both driven AND compensatory, we categorized these three types of maladaptive exercise episodes. Consistent with recent shifts away from definition maladaptive exercise in terms of frequency or duration [26, 27], we chose not to consider whether exercise was “excessive” within the current study. The type of exercise was determined by assessor judgement based on the participants’ responses to the interview questions.

#### MiFit smart band

Step count and minutes of MVPA were measured using the Mi Smart Band [28]. It collects sleep, heart rate, and daily step count data. The Mi Smart Band counts the number of steps via a 3-D accelerometer and a 3-D gyroscope, and HR is detected via a photoelectric sensor [29]. Participants wore the band over the course of the 12-week treatment. The corresponding MiFit app compiled the data, though participants were asked not to look at the app throughout the study, unless instructed by study personnel to do so (for data syncing purposes). The Mi Smart Band has demonstrated sufficient reliability and validity in measuring step count [29, 30] and heart rate [29] and high acceptability among respondents [31].

#### Data pre-processing

In line with extant literature, participants were excluded from step count and MVPA analyses if they did not wear their Mi Smart Band for more than three full valid days of the 7-day period at the pre-treatment assessment and post-treatment assessment [16, 32–35]. In addition, in line with previous work measuring step count and MVPA, a valid day of step count was defined as a 24-h period in which at least 10 waking hours of data wear time are recorded [31], and a valid full day of heart rate data was defined as a 24-h period in which at least 10 waking hours of wear time was compliant [36]. A waking hour was considered non-compliant if there was a 15-min period, where consecutive heart rate data were missing [36].

To calculate minutes of MVPA from the Mi Smart Band minute-by-minute heart rate data, target heart rate zone was calculated to determine whether each minute-by-minute heart rate data was considered a minute of moderate or vigorous physical activity. Target heart rate zone was calculated using the equations listed below based as developed by PATX, Center for Disease Control (CDC), and American College of Sports Medicine (ACSM) [37, 38]:

1. Age-predicted maximal heart rate (APMHR) = 220—age
2. Moderate Intensity Physical Activity Target heart rate (MIPATHR):
  - 64% of heart rate level = APMHR × 0.64
  - 76% of heart rate level = APMHR × 0.76
3. Vigorous Intensity Physical Activity Target heart rate (VIPATHR):
  - 77% of heart rate level = APMHR × 0.77
  - 95% of heart rate level = APMHR × 0.95

Following target heart zone calculations, minute-by-minute heart rate data were coded into minute-by-minute MVPA data. Missing data were imputed via multiple imputation analyses. The maximum amount of data imputed for a single day was three variables. Step count and MVPA data were imputed for participants that had no less than three full days of data. Of the 30 participants, 10 were excluded from the final analyses using MVPA and step count data, because they did not meet criteria for having at least three full valid days of data at the pre- or post-treatment assessment. Of the 20 participants included in the analyses using step count and MVPA data, 12 participants’ step count and MVPA data were imputed at the pre- and post-treatment assessment point. At the pre-treatment assessment point, four participants had data imputed for 1 day, six participants had data imputed for 2 days, and two participants had data imputed for 4 days. At the post-treatment assessment point four participants had data imputed for 1 day, two participants had data imputed for 2 days, two participants had data imputed for 3 days, and four participants had data imputed for 4 days.

### Statistical analyses

For Aim 1, we used paired samples *t* tests to evaluate change in self-reported amount of maladaptive exercise between individuals that were engaging in maladaptive exercise at pre-treatment. For Aim 2, we used two-way repeated measures ANOVAs to evaluate the interaction of change in the self-reported amount of adaptive exercise between the maladaptive and non-maladaptive exercise groups at pre-treatment. For Aim 3, we used two-way repeated measures ANOVAs to evaluate change in objectively measured physical activity over the duration of treatment between the maladaptive and non-maladaptive exercise groups at pre-treatment. For Aim 4, we used Pearson correlations to evaluate the relationship between changes in objectively measured physical activity and changes in EDs symptoms over the duration of treatment and Fisher’s r-to-z transformations of correlations to compare the strength of the correlations between the non-maladaptive and maladaptive exercise groups. Effect sizes were used to interpret all repeated measures ANOVAs due to small sample size and lack of statistical power. For all repeated measures ANOVAs, we used partial *η*^2^ cutoffs to determine small (0.01–0.05), moderate (0.06–0.13), and large (≥ 0.14) effect sizes. The data that support the findings of this study are available from the corresponding author upon reasonable request.

## Results

### Pre-treatment sample characteristics and descriptives

Sample characteristics are presented in Table 1 and pre-treatment maladaptive exercise episodes, adaptive exercise episodes, total step count, minutes of MVPA, and EDE descriptives are presented in Table 2. The maladaptive exercise group had higher mean scores on EDE restraint (*F*(1,28) = 31.36*, p* < 0.01) and EDE global scores (*F*(1,28) = 8.22*, p* < 0.01) compared to the non-maladaptive exercise group. There were no other significant differences between groups at pre-treatment.

**Table 1:** Sample characteristics of the non-maladaptive and maladaptive exercise groups

	Non-maladaptive exercise group		Maladaptive exercise group	
	*n*	%	*n*	%
Diagnostic presentation				
Bulimia nervosa	6	46.15	14	82.35
Binge eating disorder	7	53.85	3	17.65
Baseline characteristics				
Male	2	15.38	1	5.88
Female	11	84.62	16	94.12
White	10	76.92	13	76.47
Black	2	15.38	2	11.76
Asian	1	7.69	1	5.88
More than one race	0	0.00	1	5.88
Hispanic/Latino/Latina	1	7.69	1	5.88
Assessor-rated exercise characteristics				
Participants only engaging in adaptive exercise at pre-treatment	11	84.62	0	0
Participants not engaging in any exercise at pre-treatment	2	15.38	0	0
Participants engaging in at least one episode of maladaptive exercise 1 month prior to treatment	0	0	17	100
Treatment retention				
Dropout	1	7.69	3	17.65

**Table 2:** Pre-treatment sample descriptives of the non-maladaptive and maladaptive exercise groups

	Non-maladaptive exercise group		Maladaptive exercise group		Welch’s test		
	M	SD	M	SD	*F*	df	*p*
Baseline descriptives							
Age	37.92	13.47	36.47	11.51	2.83	1	0.76
BMI	31.26	4.62	28.19	5.36	0.10	1	0.10
Pre-treatment EDE descriptives							
Restraint subscale	1.37	1.02	3.28	0.85	29.86	1	< 0.01**
Eating concern subscale	1.80	0.88	2.18	1.35	0.85	1	0.37
Shape concern subscale	4.08	0.81	4.24	0.95	0.24	1	0.63
Weight concern subscale	3.45	0.86	3.98	1.04	2.33	1	0.14
Global score	2.67	0.46	3.42	0.84	9.53	1	< 0.01**
Total OBEs	11.92	7.35	11.18	8.10	0.07	1	0.17
Total loc episodes	24.31	10.81	24.35	12.52	0.00	1	0.99
Compensatory self-induced vomiting	1.69	3.82	5.71	11.52	1.81	1	0.19
Compensatory laxative misuse	0.08	0.28	3.29	9.79	1.83	1	0.19
Compensatory diuretic misuse	0.08	0.28	0.00	0.00	–	–	–
Compensatory fasting	2.92	3.86	3.65	3.41	0.29	1	0.60
Assessor-rated exercise descriptives							
Total number of adaptive exercise episodes	13.00	13.06	10.06	12.29	0.39	1	0.54
Average duration of total adaptive exercise episodes	33.29	31.57	24.85	24.07	0.64	1	0.43
Number of all maladaptive exercise episodes	–	–	18.12	17.17	–	–	–
All maladaptive exercise episodes average duration (minutes)	–	–	53.18	39.69	–	–	–
Number of compensatory exercise episodes	–	–	5.65	4.95	–	–	–
Compensatory exercise average duration (minutes)	–	–	74.17	67.02	–	–	–
Number of driven exercise episodes	–	–	7.24	7.51	–	–	–
Driven exercise average duration (minutes)	–	–	47.00	43.90	–	–	–
Compensatory and driven average duration (minutes)	–	–	93.64	39.18	–	–	–
Number of compensatory and driven exercise episodes	–	–	6.11	7.09	–	–	–
Pre-treatment objectively measured PA descriptives							
Mean daily steps	5209.60	1447.62	6258.56	2961.68	1.01	1	0.33
Weekly MVPA minutes	134.65	169.24	128.63	107.14	0.01	1	0.93

*EDE* Eating Disorder Examination, *MVPA* Moderate-to-Vigorous Physical Activity, *OBE* Objective Binge Episodes, *SBE* Subjective Binge Episode, *PA* Physical Activity, *CET* Compulsive Exercise Test; *p < .05; **p < .01

### Aim 1 results

#### Change in assessor-rated amount of maladaptive exercise episodes from pre- to post-treatment

See Table 3 for paired sample *t* test results demonstrating change in duration and frequency of all maladaptive exercise episodes from pre-treatment to post-treatment among the maladaptive exercise group. In line with our hypotheses, total maladaptive exercise episode duration and frequency significantly decreased from pre- to post-treatment. The duration and frequency also significantly decreased for driven exercise episodes, along with episodes that were a combination of compensatory and driven exercise; however, the duration and frequency of compensatory exercise episodes did not significantly change over the course of treatment for those that were engaging in compensatory exercise at the start of treatment.

**Table 3:** Change in average duration, frequency of total maladaptive exercise episodes within the maladaptive exercise group

		Paired Sample *t* test Results			
	Mean (SD)	*t*	df	*p*	*d*
Maladaptive exercise duration and frequency					
Pre-treatment total maladaptive exercise frequency	18.12 (17.17)	2.21	16	0.02*	0.54
Post-treatment total maladaptive exercise frequency	5.06 (14.74)				
Pre-treatment total maladaptive exercise average duration	53.18 (39.69)	3.84	16	< 0.01**	0.93
Post-treatment total maladaptive exercise average duration	15.74 (36.85)				
Pre-treatment compensatory exercise frequency	5.65 (4.95)	1.33	11	0.10	0.29
Post-treatment compensatory exercise frequency	2.83 (4.93)				
Pre-treatment compensatory exercise average duration	74.17 (67.02)	1.35	11	0.10	0.29
Post-treatment compensatory exercise average duration	46.46 (74.35)				
Pre-treatment driven exercise frequency	7.24 (7.51)	2.86	13	< 0.01**	0.68
Post-treatment driven exercise frequency	1.50 (3.82)				
Pre-treatment driven exercise average duration	47.00 (43.90)	− 2.33	13	0.04*	− 0.43
Post-treatment driven exercise average duration	15.71 (35.24)				
Pre-treatment compensatory and driven exercise frequency	6.11 (7.09)	3.29	10	< 0.01**	2.32
Post-treatment compensatory and driven exercise frequency	3.00 (–)				
Pre-treatment compensatory and driven exercise average duration	93.64 (39.18)	7.24	10	< 0.01**	− 1.20
Post-treatment compensatory and driven exercise average duration	60.00 (–)				

Duration: average minutes of exercise per exercise episode; Frequency: number of exercise episodes over 4-week period; *p < .05; **p < .01; ^a^large effect size; ^b^moderate effect size

### Aim 2 results

#### Change in assessor-rated amount of adaptive exercise episodes from pre- to post-treatment

See Table 4 for repeated measures mixed ANOVA results on change in duration and frequency of total adaptive exercise episodes from pre-treatment to post-treatment among the non-maladaptive and maladaptive exercise groups. No results were significant, which supports our hypotheses that there would be no significant change in duration and frequency of total adaptive exercise episodes from pre-treatment to post-treatment between the non-maladaptive and maladaptive exercise groups.

**Table 4:** Change in total adaptive exercise episodes duration and frequency between the non-maladaptive exercise group and the maladaptive exercise group

	Non-maladaptive exercise group	Maladaptive exercise group	Repeated measures mixed ANOVA – Interaction Result			
	M (SD)	M (SD)	*F*	*df*	*p*	η^2^
Total adaptive exercise episodes						
Pre-treatment adaptive exercise frequency	13.00 (13.06)	10.06 (12.29)	0.02	1, 28	0.90	0.00
Post-treatment adaptive exercise frequency	15.85 (10.89)	12.29 (17.17)				
Pre-treatment adaptive exercise average duration	33.29 (31.57)	24.85 (24.07)	0.02	1, 28	0.88	0.00
Post-treatment adaptive average duration	36.81 (24.84)	30.13 (31.62)				

Duration: average minutes of exercise per exercise episode; Frequency: number of exercise episodes over 4-week period; *p < .05; **p < .01; ^a^large effect size; ^b^moderate effect size

### Aim 3 results

#### Interaction between change in total step count and minutes of MVPA from pre- to post-treatment between the maladaptive and non-maladaptive exercise groups

Results of the interaction of change in total step count and minutes of MVPA across treatment between both groups are presented in Table 5. Contrary to our hypotheses, there were no significant differences when comparing change in step count and minutes of MVPA from pre- to post-treatment among the non-maladaptive and adaptive exercise groups. However, although not significant, there was a moderate effect size of the interaction of minutes of MVPA from pre- to post-treatment between groups, suggesting the minutes of MVPA increased more from pre- to post-treatment in the non-maladaptive exercise group compared to the maladaptive exercise group.

**Table 5:** Change in objectively measured physical activity from pre- to post-treatment among the total sample, the non-maladaptive exercise group, and the maladaptive exercise group

	Total Sample	Non-maladaptive exercise group	Maladaptive exercise group	Repeated measures mixed ANOVA—interaction result			
	M (SD)	M (SD)	M (SD)	*F*	df	*p*	*η*^2^
Objectively measured step count				0.19	1	0.67	0.02
Per-treatment mean daily steps	5734.08 (2331.77)	5209.60 (1447.62)	6258.56 (2961.68)				
Post-treatment mean daily steps	6771.93 (2344.22)	6081.31 (1172.03)	7462.54 (3027.84)				
*Objectively Measured MVPA*				2.06	1	0.15	0.12^b^
Pre-treatment weekly MVPA minutes	131.64 (137.90)	134.65 (169.24)	128.63 (107.14)				
Post-treatment weekly MVPA minutes	132.43 (130.28)	155.09 (171.81)	123.19 (75.80)				

Mean Daily Step Count: mean number of daily steps over a 7-day period; *MVPA* Minutes of Moderate to Vigorous Physical Activity; Weekly MVPA Minutes: total minutes of MVPA over a 7-day period; *p < .05; **p < .01; ^a^large effect size; ^b^moderate effect size

### Aim 4 results

#### The relationship between change in step count, minutes of MVPA, and ED symptoms from pre- to post-treatment between the maladaptive and non-maladaptive exercise groups

See Table 6 for complete results of the Pearson correlations and Fisher’s r-to-z transformation of correlations. There were no significant correlations between change in ED symptomatology and step count or minutes of MVPA from pre- to post-treatment for the non-maladaptive and maladaptive exercise group. This supports our hypotheses for the maladaptive group but not the non-maladaptive exercise group. Analyses using Fisher’s r-to-z transformation of correlations, which assessed changes in objectively measured physical activity and changes in EDs symptoms over the duration of treatment, indicated several correlations were significantly different between the maladaptive and non-maladaptive exercise groups at the 0.05 level (Z >  ± 1.96, two-tailed). Specifically, there were significant differences in change in minutes of MVPA from pre- to post-treatment was correlated with change in self-reported objective binge episodes (OBEs) and the EDE global score from pre- to post-treatment. The comparative strength of these correlations suggests that among the maladaptive exercise group, decreases in minutes of MVPA from pre- to post-treatment was related to decreases in self-reported OBEs and decreases in the global EDE score from pre- to post-treatment, which was contrary to our hypothesis.

**Table 6:** Comparing correlational strength of change in eating disorder symptoms, average daily step count, and total weekly minutes of MVPA over the course of treatment among those in the non-maladaptive and maladaptive exercise groups

	Average daily step count change score			Total weekly minutes of MVPA change score		
	Non-maladaptive *r*	Maladaptive* r*	*Z-*difference	Non-Maladaptive *r*	Maladaptive* r*	*Z*-difference
EDE symptoms						
EDE OBE frequency change score	0.09	0.25	− 0.40	0.53	− 0.33	2.25*
EDE LOC episode frequency change score	− 0.06	0.18	− 0.58	− 0.18	0.18	− 0.88
EDE compensatory behavior frequency change score	0.04	0.05	− 0.02	− 0.18	− 0.02	− 0.39
Global score change score	− 0.34	0.20	− 1.34	− 0.23	0.42	− 1.65*

*p < .05; **p < .01

## Discussion

The current study used a multi-modal approach to examine how both assessor-rated type and amount of maladaptive and adaptive exercise, and objectively measured physical activity changed over the course of a 12-week CBT treatment program in 30 individuals with transdiagnostic binge eating and restrictive eating.

Individuals that engaged in maladaptive exercise at the start of treatment reported engaging in a variable amounts of driven exercise, compensatory exercise, and driven and compensatory exercise. At post-treatment, no individuals reported engaging in driven or compensatory and driven exercise episodes. Overall and in line with previous research, CBT was very effective at reducing the overall amount of maladaptive exercise [21–23]. CBT also significantly reduced the amount of driven exercise and driven and compensatory exercise episodes but not exercise episodes that were exclusively compensatory. Based on clinical observations, this result may be due to individuals relying too heavily on compensatory exercise as a safety behavior. Furthermore, compared to driven exercise, current CBT treatments for EDs may not be not be adequately equipped to reduce compensatory exercise. Although CBT was effective at reducing maladaptive exercise episodes overall, we can conclude CBT may benefit from more specific guidance for therapists on how to address compensatory exercise, and it is possible driven exercise and compensatory exercise may need to be addressed differently in CBT.

The amount of total adaptive exercise was similar between groups at both pre- and post-treatment, and the total amount of adaptive exercise did not notably increase from pre- to post-treatment between groups. In the maladaptive exercise group, all participants were engaging in adaptive exercise episodes at the end of treatment and in the non-maladaptive exercise group, more individuals were engaging in adaptive exercise at the end of treatment. CBT is not designed to promote and increase adaptive exercise in individuals with EDs, and this investigation suggests that CBT for EDs does not incidentally increase the amount of adaptive exercise for individuals with transdiagnostic binge eating and restrictive eating, regardless of if they entered treatment engaging in maladaptive exercise. Given the positive benefits individuals with EDs can experience from engaging in adaptive exercise, future research may benefit from continuing to explore whether intentionally promoting adaptive exercise in a CBT treatment for EDs could increase the overall amount of exercise adaptive exercise for individuals with EDs that do and do not engage in maladaptive exercise at the start of treatment.

Our findings from measuring physical activity objectively are in line with the few other studies that have also measured physical activity objectively in EDs. The recorded step counts at both pre- and post-treatment for both groups were comparable our previous study that objectively measured step count in individuals with transdiagnostic binge eating [3]. In addition, the step count results suggest individuals with transdiagnostic binge eating have an average step count similar to healthy adults in the United States (5000–6000 steps), which is a step count range indicating an individual has relatively low activity levels [39]. We can conclude individuals in both groups did not increase their overall levels of physical activity (i.e., take stairs instead of elevator, reaching > 8000 steps on average per day) over the course of treatment. Interestingly, although maladaptive exercise did significantly decrease for individuals in the maladaptive exercise group, their step count did not significantly reduce. It is possible that their maladaptive exercise episodes were replaced with either adaptive exercise or more general physical activity (i.e., take stairs instead of elevator, reaching > 8000 steps on average per day). Furthermore, data collected as part of this project that is beyond the scope of the current study suggests that individuals in the maladaptive exercise group did not significantly change the type (e.g., running, ball sports, walking, cycling, etc.) of overall exercise they engaged in during the course of treatment, which supports the possibility that their exercise episodes were replaced with either adaptive exercise or more general physical activity. Future research should more closely assess this possibility.

In regard to minutes of MVPA, 60% of individuals in the non-maladaptive exercise group and 50% in the maladaptive exercise group were not meeting the current physical activity recommendations from the CDC and the ACSM (minimum of 150–300 min of MVPA per week) at the start of treatment [40]. At the end of treatment, 50% of individuals in the non-maladaptive exercise group and 60% in the maladaptive exercise group were not meeting the current physical activity recommendations from the CDC and the ACSM. In addition, the non-maladaptive exercise group was engaging in significantly more minutes of MVPA at the end of treatment compared to the maladaptive exercise group. The percentages of groups at both pre- and post-treatment who were meeting physical activity recommendations are comparable to two other studies that have objectively measured MVPA in individuals with transdiagnostic binge eating [3, 22, 23] and one study that used self-report measurement to assess whether individuals with binge eating met the CDC and ACSM physical activity recommendations [41]. Clinically, this result suggests that if clinicians are interested in increasing adaptive physical activity in individuals with EDs and having more individuals reach the CDC and ACSM physical activity recommendations, employing an additional treatment to CBT (or an entirely different treatment) may be warranted.

Increases in MVPA and step count were not associated with improvements in ED symptoms for both groups. Clinically, this may suggest increasing physical activity may not improve ED outcomes. However, it is also possible the observed increase in physical activity led to other types of benefits commonly associated with physical activity that were not measured in the current study, such as physiological, psychological, and social benefits. Interestingly, we found decreases in minutes of MVPA in the maladaptive exercise group were related to decreases in EDE symptomatology and binge eating frequency. This result may suggest decreases in minutes of MVPA is a result of decreases in minutes of maladaptive exercise, and such decreases may help improve overall ED symptomatology. Furthermore, this finding may also suggest that when individuals in the maladaptive exercise group were no longer binge eating, they no longer felt the need to engage in compensatory exercise, which indicates that engaging in exercise behaviors during ED recovery may not be advantageous for all presentations of ED symptoms.

Altogether, future studies should replicate these findings in a larger more representative sample outside of the COVID-19 pandemic, continue to use multimodal assessment of physical activity, assess the effects of using fitness trackers in ED treatments to promote positive change in ED symptoms and increase adaptive physical activity, and consider using ecological momentary assessment in conjunction with fitness trackers to determine when objectively measured physical activity is or is not maladaptive. Overall, the many novelties of the current study have allowed for greater insight into how the amount of maladaptive exercise, adaptive exercise, and objectively measured physical activity changed over the course of a CBT treatment for individuals with transdiagnostic binge eating and restrictive eating that do and do not start CBT engaging in maladaptive exercise. The present study also provides insight into possible ways to adequately address adaptive and maladaptive exercise and overall levels of physical activity in individuals with transdiagnostic binge eating and restrictive eating moving forward.

## Strengths and limits

Despite the many important findings and implications from the current study, there are several notable limitations. First, the sample was small, mostly white, most individuals identified as female, and individuals with Anorexia Nervosa (AN) were excluded from the study. Future research should aim to explore the current research questions in a more diverse sample and in individuals with AN given that maladaptive exercise is a highly prevalent symptom in individuals with AN [42]. Another limitation was that both groups had unequal sample sizes. In addition, a portion of the objectively measured physical activity data was imputed via a regression imputation, which can lead to biased parameter estimates. Imputed data also suggest that future research should be cautious of the feasibility and acceptability of having participants wear wearable fitness trackers throughout the duration of treatment. Furthermore, the assessor-rated adaptive and maladaptive exercise was measured at the baseline and post-treatment assessment and the objectively measured physical activity were assessed during the first week and last week of treatment, which may have impacted the increasing or decreasing of some exercise physical activity data. This study also collected minimal cognitive components of maladaptive exercise. Future research should aim to collect a robust set of data that include both cognitive and objective measures of maladaptive exercise, as some research suggests maladaptive exercise is a primarily cognitive construct [43]. It is also important to highlight that this study lacked power, making it difficult to interpret the significance and effect sizes of many of the results. Furthermore, although the modified EDE is a novel way to assess adaptive versus maladaptive exercise and is based on sound structure, the modified version is not well-validated. Finally, all of the data in the current study was collected during the COVID-19 pandemic, which may be a limitation as some research has suggested many adults became more sedentary throughout the pandemic and may have not had access to their normal physical activity routine (e.g., working out in fitness center, attending in-person fitness classes) [44, 45]. Alternatively, some research has demonstrated that many adults with [46] and without eating disorders [47] became more physically active throughout the COVID-19 pandemic.

## Data Availability

The data sets generated during and/or analyzed during the current study are available from the corresponding author on reasonable request.

## References

[1] Meyer C, Taranis L, Goodwin H, Haycraft E (2011). Compulsive exercise and eating disorders. Eur Eat Disord Rev.

[2] Holland LA, Brown TA, Keel PK (2014). Defining features of unhealthy exercise associated with disordered eating and eating disorder diagnoses. Psychol Sport Exerc.

[3] Wons OB, Michael ML, Lin M, Juarascio AS (2020) Characterizing rates of physical activity in individuals with binge eating disorder using wearable sensor technologies and clinical interviews. Eur Eating Disord Rev. 10.1002/erv.2811 PMC8087155 33247869

[4] Hausenblas HA, Downs DS (2002). Exercise dependence: a systematic review. Psychol Sport Exerc.

[5] Lease HJ, Bond MJ (2013). Correspondence between alternate measures of maladaptive exercise, and their associations with disordered eating symptomatology. J Behav Addict.

[6] Stiles-Shields EC, Goldschmidt AB, Boepple L, Glunz C, Le Grange D (2011). Driven exercise among treatment-seeking youth with eating disorders. Eat Behav.

[7] American Psychiatric Association (2015). Feeding and eating disorders: DSM-5® selections.

[8] Márquez S, Molinero O (2013). Energy availability, menstrual dysfunction and bone health in sports; an overview of the female athlete triad. Nutr Hosp.

[9] Melissa R, Lama M, Laurence K, Sylvie B, Jeanne D, Odile V, Nathalie G (2020). Physical Activity in Eating Disorders: A Systematic Review. Nutrients.

[10] Stiles-Shields C, DclinPsy BB, Lock J, Le Grange D (2015). The effect of driven exercise on treatment outcomes for adolescents with anorexia and bulimia nervosa. Int J Eat Disord.

[11] Calogero R, Pedrotty K (2004). The practice and process of healthy exercise: an investigation of the treatment of exercise abuse in women with eating disorders. Eating Disord.

[12] De Young KP, Anderson DA (2010). The importance of the function of exercise in the relationship between obligatory exercise and eating and body image concerns. Eat Behav.

[13] Kerrigan SG, Lydecker JA, Grilo CM (2019). Associations between physical activity and eating-disorder psychopathology among individuals categorised with binge-eating disorder and bulimia nervosa. Int J Clin Pract.

[14] Lampe EW, Forman EM, Juarascio AS, Manasse SM (2021). Feasibility, acceptability, and preliminary target engagement of a healthy physical activity promotion intervention for bulimia nervosa: development and evaluation via case series design. Cogn Behav Pract.

[15] Cook B, Wonderlich SA, Mitchell J, Thompson R, Sherman R, Mccallum K (2016). Exercise in eating disorders treatment: systematic review and proposal of guidelines. Med Sci Sports Exerc.

[16] Mathisen TF (2018) A randomized controlled trial of physical exercise-and dietary therapy versus cognitive behavior therapy: treatment effects for women with bulimia nervosa or binge eating disorder.

[17] Vancampfort D, Probst M, Adriaens A, Pieters G, De Hert M, Stubbs B, Soundy A, Vanderlinden J (2014). Changes in physical activity, physical fitness, self-perception and quality of life following a 6-month physical activity counseling and cognitive behavioral therapy program in outpatients with binge eating disorder. Psychiatry Res.

[18] National Institute for Health and Care Excellence (NICE) (2017). NG69 eating disorders: recognition and treatment.

[19] Wilson GT (2005). Psychological treatment of eating disorders. Annu Rev Clin Psychol.

[20] Fairburn CG (2008). Cognitive behavior therapy and eating disorders.

[21] Byrne SM, Fursland A, Allen KL, Watson H (2011). The effectiveness of enhanced cognitive behavioural therapy for eating disorders: an open trial. Behav Res Ther.

[22] Mathisen TF, Bratland-Sanda S, Rosenvinge JH, Friborg O, Pettersen G, Vrabel KA, Sundgot-Borgen J (2018). Treatment effects on compulsive exercise and physical activity in eating disorders. J Eat Disord.

[23] Mathisen TF, Rosenvinge JH, Friborg O, Pettersen G, Stensrud T, Hansen BH, Underhaug KE, Teinung E, Vrabel K, Svendsen M, Bratland-Sanda S, Sundgot-Borgen J (2018). Body composition and physical fitness in women with bulimia nervosa or binge-eating disorder. Int J Eat Disord.

[24] Pendleton VR, Goodrick GK, Poston WSC, Reeves RS, Foreyt JP (2002). Exercise augments the effects of cognitive-behavioral therapy in the treatment of binge eating. Int J Eat Disord.

[25] Cooper Z, Fairburn C (1987). The eating disorder examination: a semi-structured interview for the assessment of the specific psychopathology of eating disorders. Int J Eat Disord.

[26] Adkins EC, Keel PK (2005). Does “excessive” or “compulsive” best describe exercise as a symptom of bulimia nervosa?. Int J Eat Disord.

[27] Mond and Gorrell. (2021). “Excessive exercise” in eating disorders research: problems of definition and perspective. Springer. pp 1–4 10.1007/s40519-020-01075-3 PMC8068596 33389704

[28] Mi Smart Band 4. Mi Smart Band 4丨Xiaomi UK丨Mi.com - Xiaomi UK. (n.d.). Retrieved August 23, 2022, from https://www.mi.com/uk/mi-smart-band-4/

[29] de la Casa Pérez A, Latorre Román P, Muñoz Jiménez M, Lucena Zurita M, Laredo Aguilera JA, Párraga Montilla JA, Cabrera Linares JC (2022) Is the Xiaomi Mi Band 4 an Accuracy Tool for Measuring Health-Related Parameters in Adults and Older People? An Original Validation Study. Int J Environ Res Public Health. 10.3390/ijerph19031593 10.3390/ijerph19031593 PMC8834826 35162615

[30] Ricchio K, Lyter-Antonneau P, Palao JM (2018). Reliability of fitness trackers at different prices for measuring steps and heart rate: a pilot study. Cent Eur J Sport Sci Med.

[31] Tam MK, Cheung SY (2019). Validation of consumer wearable activity tracker as step measurement in free-living conditions. Finnish J eHealth eWelfare.

[32] Del Giudice F, Glover F, Belladelli F, De Berardinis E, Sciarra A, Salciccia S, Eisenberg ML (2021). Association of daily step count and serum testosterone among men in the United States. Endocrine.

[33] Norris M, Anderson R, Motl RW, Hayes S, Coote S (2017). Minimum number of days required for a reliable estimate of daily step count and energy expenditure, in people with MS who walk unaided. Gait Posture.

[34] O'Brien MW, Wojcik WR, D'Entremont L, Fowles JR (2018). Validation of the PiezoRx® step count and moderate to vigorous physical activity times in free living conditions in adults: a pilot study. Int J Exerc Sci.

[35] Yao J, Tan CS, Lim N, Tan J, Chen C, Müller-Riemenschneider F (2021). Number of daily measurements needed to estimate habitual step count levels using wrist-worn trackers and smartphones in 212,048 adults. Sci Rep.

[36] Brazendale K, Decker L, Hunt ET, Perry MW, Brazendale AB, Weaver RG, Beets MW (2019). Validity and wearability of consumer-based fitness trackers in free-living children. Int J Exerc Sci.

[37] Faust L, Wang C, Hachen D, Lizardo O, Chawla NV (2019). Physical activity trend extraction: a framework for extracting moderate-vigorous physical activity trends from wearable fitness tracker data. JMIR Mhealth Uhealth.

[38] Scheid JL, O’Donnell E (2019). Revisiting heart rate target zones through the lens of wearable technology. ACSM's Health Fitness J.

[39] Hoeger WWK, Bond L, Ransdell L, Shimon JM, Merugu S (2008). ONE-mile step count at walking and running speeds. ACSM's Health Fitness J.

[40] Piercy KL, Troiano RP, Ballard RM, Carlson SA, Fulton JE, Galuska DA, George SM, Olson RD (2018). The physical activity guidelines for Americans. JAMA.

[41] Carr MM, Lydecker JA, White MA, Grilo CM (2019). Examining physical activity and correlates in adults with healthy weight, overweight/obesity, or binge-eating disorder. Int J Eat Disord.

[42] Trott M, Jackson SE, Firth J, Jacob L, Grabovac I, Mistry A, Stubbs B, Smith L (2021). A comparative meta-analysis of the prevalence of exercise addiction in adults with and without indicated eating disorders. Eating Weight Disord Stud Anorexia Bulimia Obesity.

[43] Goodwin H, Haycraft E, Meyer C (2016). Disordered eating, compulsive exercise, and sport participation in a UK adolescent sample. Eur Eat Disord Rev.

[44] Alomari MA, Khabour OF, Alzoubi KH (2020). Changes in physical activity and sedentary behavior amid confinement: the BKSQ-COVID-19 Project. Risk Manag Healthc Policy.

[45] Zheng C, Huang WY, Sheridan S, Sit CH-P, Chen X-K, Wong SH-S (2020). COVID-19 pandemic brings a sedentary lifestyle in young adults: a cross-sectional and longitudinal study. Int J Environ Res Public Health.

[46] Haghshomar M, Shobeiri P, Brand S, Rossell SL, Akhavan Malayeri A, Rezaei N (2022). Changes of symptoms of eating disorders (ED) and their related psychological health issues during the COVID-19 pandemic: a systematic review and meta-analysis. J Eat Disord.

[47] León-Zarceño E, Moreno-Tenas A, Boix Vilella S, García-Naveira A, Serrano-Rosa MA (2021). Habits and psychological factors associated with changes in physical activity due to COVID-19 confinement. Front Psychol.

